# Examining Critical Reflection as a Mediator Between School Racial Climate Experiences and Anti‐Racist Action

**DOI:** 10.1111/jora.12778

**Published:** 2022-06-16

**Authors:** Alexandrea R. Golden, Christy M. Byrd

**Affiliations:** ^1^ Cleveland State University; ^2^ North Carolina State University

## Abstract

Little is known about how different school racial climate experiences influence the critical reflection and subsequent critical action behaviors of racially minoritized youth. Therefore, the current study examined how critical reflection mediated the relationship between school racial climate profiles and critical action behaviors. Participants were 559 Black and Latinx adolescents, aged 13–17 who completed an online survey. Results indicated that critical reflection significantly mediated the relationships between interpersonal interactions (i.e., equal status) and anti‐racist critical action behaviors. Similarly, the relationships between school racial socialization messages (i.e., cultural and critical consciousness socialization) and anti‐racist critical action behaviors were also mediated by critical reflection. Findings have implications for how dimensions of the school racial climate differentially relate to racially minoritized youth's critical consciousness.

The school environment is a critical space for developing youth into productive civic actors in our democratic society. This is important both for the political advancement of our nation as youth move into their careers and for promoting a more socially and racially equitable society and reducing disparities. Although explicit teaching of history, civics, and critical thinking is consequential for the civic development of youth, the racial interactions and practices within the school environment (i.e., school racial climate) may also influence youth's understanding of systemic inequities and encourage civic action (Leath & Chavous, 2017; Pinedo et al., 2021). Experiencing racial inequities through interpersonal interactions, the application of policies and practices, and presence, or lack thereof, of representation of diverse cultures in the curriculum and school environment in general (i.e., school racial climate) can have detrimental consequences for students of color via maladaptive coping (Golden et al., 2018; Johnston‐Goodstar & VeLure Roholt, 2017). Critical consciousness may be an alternative pathway to adaptive coping for youth of color (Hope & Spencer, 2017) experiencing racial inequities that can have greater implications for their development.

Critical consciousness, the process by which youth come to develop an awareness of forms of oppression such as racism and act against it (Watts et al., 1999, 2011), has been identified as a coping tool for racially minoritized youth experiencing racial inequities (Hope & Spencer, 2017). Theorists suggest that critical reflection, that is, youth's understanding of the impacts of structural oppression, may increase as a result of racial experiences such as racial discrimination and discussions about racial inequities (Anyiwo et al., 2018). This critical reflection may then lead to youth engaging in individual and collective critical action to resist and dismantle racist systems (Watts et al., 2011). Nevertheless, it remains unclear how different aspects of the school racial climate relate to youth's anti‐racist critical action behaviors and whether critical reflection explains this relationship. To address this gap, the current study explored how adolescents' experiences of their school racial climate are associated with their critical action through critical reflection.

## CRITICAL CONSCIOUSNESS

Critical consciousness theory (Freire, 1970; Jemal, 2017; Watts et al., 2011) provides a conceptualization of the process by which marginalized individuals gain awareness of their oppressive conditions and examine the factors that contribute to those oppressive conditions (i.e., critical reflection) and further act to change these conditions and dismantle oppressive systems (i.e., critical action). Freire's (1970) original conceptualization of critical consciousness focuses on praxis—the reciprocal relationship between critical reflection and critical action—to highlight how “critical understanding leads to critical action” (Freire, 1970, p. 42).

While critical consciousness is important for all adolescents due to their increasing awareness of the social issues occurring in the world around them and their civic responsibilities to contribute to a democratic society through behaviors such as volunteering and voting (i.e., civic development; Flanagan & Levine, 2010), it is particularly important for racially minoritized youth. For youth of color, this awareness and sense of responsibility is compounded by their expanding understanding of racial inequities and their direct and indirect experiences of racial discrimination (Rubin, 2007). As youth of color engage in the process of critical reflection to understand the root causes of social issues, such as racism, they will be more inclined to act upon these issues. Aligned with the original conceptualization of praxis, the current study examined the relationship between critical reflection and critical action.

Recent studies of critical consciousness among racially minoritized youth provide a more nuanced understanding of how these youth analyze and respond to racial inequities and highlight the importance of nontraditional acts of resistance for racially minoritized youth (Golden et al., 2021; Hope, Gugwor, et al., 2019; Tyler et al., 2020). These studies resist the long‐standing narrative that youth of color are less engaged in civic engagement behaviors and amplify the diverse experiences of racially marginalized youth. Youth of color engage in various critical action behaviors aimed at resisting oppression at different levels including influencing changes in individuals, communities, and policies (Aldana et al., 2019). For example, racially marginalized youth may choose to disrupt oppressive rhetoric or address disparaging racial remarks made by others (Aldana et al., 2019; Hope, Pender, et al., 2019) to combat racism in an individual capacity. They may also choose to participate in community organizations (e.g., NAACP, local organizations) that seek to give back and impact change specifically in their communities. To honor the range of racially minoritized youth's expressions of critical action behaviors and examine behaviors specific to the racial challenges that racially minoritized youth experiences, this study examined traditional, nontraditional, and multisystemic critical action behaviors using the Anti‐racism Action Scale (Aldana et al., 2019). The Anti‐racism Action Scale assesses the various ways in which youth challenge racism through anti‐racist critical action behaviors and was informed by the experiences of racially minoritized youth.

## SCHOOL AS A DEVELOPMENTAL CONTEXT FOR CRITICAL CONSCIOUSNESS

Given the goal of cultivating informed and civically engaged members of a democratic society, the educational context is an important space for developing critical consciousness among adolescents (Heberle et al., 2020; Seider et al., 2017, 2020). In addition to their role of delivering core curricular content and promoting critical thinking skills, schools can also promote youths' critical consciousness through the school racial climate—the patterns of norms, values, messages, and interactions related to race and culture (Byrd, 2015). More specifically, messages including those highlighting diversity and social justice and experiences of racial inequities within the school (i.e., interpersonal interactions) may be associated with how youth analyze and act against racism (Anyiwo et al., 2018; Mathews et al., 2019; Tyler et al., 2020). The following sections will further discuss the ways that messages about race and racism (school racial socialization), and school‐based racial inequities may promote critical consciousness development.

### School Racial Socialization

Although the relationship between racial socialization and critical consciousness development has primarily been examined in parent‐to‐child communications, studies have located the school environment as an important source of racial socialization for youth of color (Byrd, 2015, 2017). Schools have not been studied as sites of racial socialization in part because, historically, course content in schools has overemphasized mainstream culture, while diverse cultures are underrepresented in course materials (Hope et al., 2015; Woodson, 2015). This remains an ongoing challenge despite scholars' emphasis on the importance of messages about race through the inclusion, or lack thereof, of diverse cultures and discussion of social injustices in the classroom, and the school environment more broadly, for the development of racially minoritized youth (Byrd & Hope, 2020; Matthews & López, 2019; Stowe, 2017). However, messages about race and racism at school can influence Black and Latinx youth's conceptualization of the individual‐ and systemic‐level factors that contribute to racial disparities by alerting youth to the potential for experiencing racism, by promoting coping skills and pride in their culture, and by helping youth reflect on their similarities and differences to those from other marginalized groups (Bañales et al., 2020; Hope & Bañales, 2019; Lozada et al., 2017).

In the current study, we focused on two types of school racial socialization messages (Byrd, 2017): critical consciousness socialization and cultural socialization. Critical consciousness socialization describes messages from teachers about racial inequality and social justice. Given work in the parental literature relating preparation for bias (messages anticipating discrimination) to critical reflection (Wang et al., 2020), scholars have expected that greater perceptions of critical consciousness socialization would be associated with greater critical consciousness in adolescents. In their work, Bañales et al. (2019) did not find a direct relation between critical consciousness socialization and critical reflection, but they did find that critical consciousness socialization was indirectly related to interpersonal and communal action through anger toward social injustices.

The current study attempted to replicate the findings of Bañales et al. (2019) by examining how critical consciousness socialization is related to anti‐racist action. Further, we also explored cultural socialization messages based on work that suggests that the inclusion and discussion of diverse cultures, historical events, and controversial topics increase youth's awareness of structural oppression (i.e., critical reflection), which may lead to subsequent increases in their critical action (Bañales et al., 2019; Godfrey & Grayman, 2014; Seider et al., 2020). For example, Godfrey and Grayman (2014) found that for racially minoritized high school students, a classroom climate that encouraged diverse opinions on political and social issues was associated with youth's participation in community‐based action, but not their analysis of inequities. Further, Seider et al. (2020) found that the progressive and no‐excuses schools in their study varied in how they approached student–teacher dynamics, but in both types of schools, an emphasis on sociopolitical engagement was associated with higher critical reflection and critical action for students. Finally, both parental and school cultural socialization messages tend to be related to positive outcomes (Byrd, 2017; Saleem & Byrd, 2021; Wang et al., 2020).

### School‐Based Racial Inequities

The final indicator of school racial climate is school‐based racial inequities. Experiences of racial discrimination have been significantly associated with youth's increased critical consciousness (Hope et al., 2020; Pinedo et al., 2021; White‐Johnson, 2012), yet much of this literature has focused on racial inequities outside of the school environment. While schools are opportunity structures for fostering critical consciousness (Watts & Flanagan, 2007), the practices and policies within schools often reconstruct the inequitable policies and structures experienced by marginalized youth outside of the school environment (Seider et al., 2020). For example, youth of color may experience racial discrimination in their interactions with other students and teachers and administrators (Leath et al., 2019). Additionally, they may have to contend with negative stereotypes and associated behaviors, such as believing that Black students are not as smart, and resultantly receiving lower expectations or less feedback from teachers (Hope et al., 2015). They may also be aware of and need to cope with inequitable enforcement of policies such as unfair discipline practices (Gopalan & Nelson, 2019; Wegmann & Smith, 2019).

Racial inequities in the school environment may increase racially minoritized youth's critical consciousness by prompting them to seek a greater understanding of why these experiences occurred, thus leading them to racialized explanations and a greater understanding of the systemic issue of racism (Mathews et al., 2019). Subsequently, youth may become civically involved to impact change on these issues. For example, Black and Latinx youth are keenly aware of the inequitable enforcement of rules (e.g., dress code) and disciplinary practices within the school that result in the overrepresentation of these youth in office referrals and school suspensions (Kaufman et al., 2010; Kelly, 2018; Ruck & Wortley, 2002). Youth's identification of systemic inequities within the school environment has been associated with increased activism (Leath & Chavous, 2017), even years beyond these experiences. This was demonstrated in a sample of Black and Latinx college students, where experiences of racial discrimination during high school significantly predicted increases in participants' critical action during their freshman and sophomore years of college (Pinedo et al., 2021).

Although school‐based inequities and school racial socialization messages have been identified as potentially important influences of critical consciousness development, few studies have empirically examined these relationships. Further, existing studies are mixed on the relations between school racial climate experiences and youth's critical consciousness (Lozada et al., 2017). The current study helps elucidate the relations between school‐based inequities and critical consciousness in youth of color.

## The Current Study

The current study was guided by school racial climate and critical consciousness theories. Scholars suggest that increases in critical reflection may lead to increased critical action. Although some scholars have highlighted the importance of the school environment in promoting the development of critical consciousness, including both critical reflection and critical action, there is a dearth in research examining these relationships. Moreover, few studies examine the racialized school experiences that contribute to the critical consciousness development of racially minoritized youth. Racially minoritized youth's perceptions of the school racial climate highlight not only their awareness of differences in perspectives and experiences of students and the function of race in those differences, but may also highlight the transition from youth's awareness of structural oppression to their action toward impacting changes across systems. Therefore, in the current study we examined how school racial climate perceptions are associated with critical reflection and critical action.

Given that “critical understanding leads to critical action” (Freire, 1970, p. 42; see also Hope et al., 2020; Tyler et al., 2020), we also tested whether critical reflection served as a mediator of the relations between racial climate and critical action. The research question was: Are perceptions of school racial climate, particularly equal status, cultural socialization, and critical consciousness socialization, associated with different forms of critical action through critical reflection? We hypothesized that school racial climate would be significantly related to critical action through critical reflection. Despite competing literature, we believe that it is youth's awareness of structural racial inequities due to their experiences that prompt their engagement in anti‐racist critical action to dismantle those oppressive systems.

## METHOD

### Participants and Procedure

Participants included 559 Black and Latinx adolescents, aged 13–17 (*M* = 15.27, *SD* = 1.58) recruited from across the United States. Fifty percent of participants identified as female. Procedures were approved by the Institutional Review Board at the second author's university. Participants were recruited through Qualtrics Panels, an online survey delivery service that researchers can use to recruit study participants. Parents were recruited through an email invitation that included the expected duration of the study and the type of incentive available for participation. Parents completed a screening survey; if they had a child between the ages of 13 and 17 who attended public or private school, the parent was asked to give consent for their child to complete the study. The parent was then asked to have their child complete the rest of the survey online. Adolescents then completed demographic information; those who identified as White, African American, Asian American, or Latinx were allowed to continue to the rest of the survey until quotas for their ethnic‐racial group (approximately 250 in each group) were filled. Only Black and Latinx participants are included in the current sample. Parents were compensated in credit that they could redeem through Qualtrics for gift cards and other awards.

### Measures

#### School racial climate

School racial climate perceptions were assessed using the School Climate for Diversity Scale—Secondary, which has been validated in diverse samples of adolescents (see Byrd, 2017). The subscales were equal status (four items, *α* = .87; e.g., “The principal(s) treat students of all races/ethnicities fairly”), cultural socialization (five items, *α* = .88; e.g., “At your school, you have chances to learn about the history and traditions of your culture”), and critical consciousness socialization (seven items, *α* = .80; e.g., “Teachers teach about racial inequality in the United States”). All were on a response scale of 1 (*not at all true*) to 5 (*completely true*).

#### Critical consciousness

Critical reflection was measured using the Critical Consciousness Scale (Diemer et al., 2017), which is comprised of eight items (*α* = .94; e.g., “Certain racial or ethnic groups have fewer chances to get a good high school education”). The response scale was 1 (*strongly disagree*) to 5 (*strongly agree*). Critical action was assessed using the Anti‐racism Action Scale (Aldana et al., 2019). This measure is comprised of three subscales including interpersonal action (five items; *α* = .85; e.g., “Challenged or checked a friend who uses a racial slur or makes a racial joke”), communal action (four items; *α* = .80; e.g., “Participated in a leadership group or committee working on issues related to race, ethnicity, discrimination, and/or segregation”), and political action (seven items; *α* = .81; e.g., “Called/written/emailed an elected official (i.e. city council, mayor, legislator)”). Each item was answered (1) “Yes” or (0) “No.” Scores were summed so that higher scores indicated more action.

### Data Analysis

**Figure 1:**
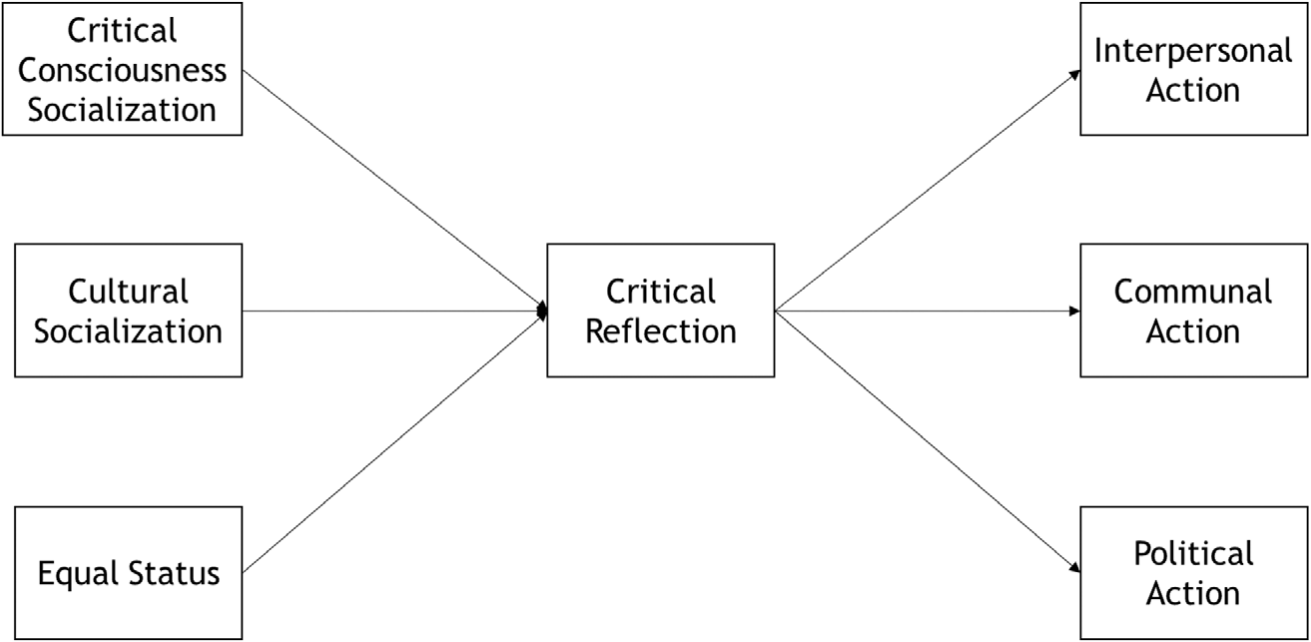
Analysis model of school racial socialization associations with critical action through critical reflection.

The analyses were conducted using a path analysis in Stata 15.1 (StataCorp LLC, College Station, Texas, TX, USA) with full information maximum‐likelihood estimation. We estimated the relations between equal status, cultural socialization, and critical consciousness socialization and interpersonal, communal, and political action and estimated the indirect effects of critical reflection as a mediator (see Figure 1). Age, gender, and race (Black or not) were control variables. Bootstrapped confidence intervals for the indirect effect were estimated to determine the significance of the mediation. Some statisticians have expressed concern about testing for mediation in cross‐sectional data (e.g., Maxwell et al., 2011). We agree with Hayes (2018) that no study can capture the entire complexity of a process; however, statistical methods such as mediation are useful tools in attempting to make sense of reality.

## RESULTS

**Figure 2:**
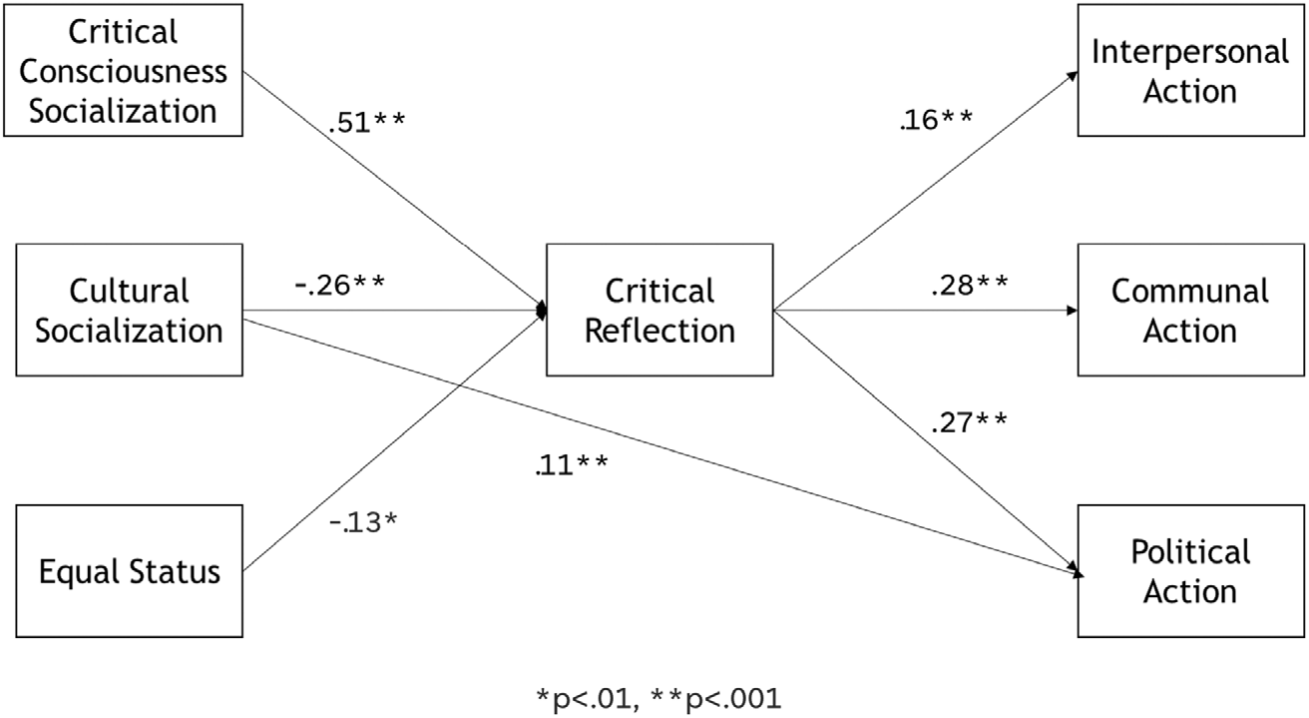
Final model of school racial socialization associations with critical action through critical reflection.

Descriptive statistics and correlations are shown in Table 1. Model fit was acceptable, but we examined modification indices to determine opportunities to improve model fit and found that the model would be improved by adding a direct effect between cultural socialization and critical political action (Figure 2). The final model fit was chi‐square (8, *N* = 559) = 44.88, *p* < .001, RMSEA = .091, CFI = .942. The standardized model coefficients can be seen in Tables 2 and 3.

**Table 1:** Means and Standard Deviations for Study Variables

Variable	Mean	*SD*	1	2	3	4	5	6
1. Equal status	3.70	0.99						
2. Cultural socialization	3.09	1.03	.54***					
3. Critical consciousness socialization	3.02	0.97	.46***	.69***				
4. Critical reflection	2.80	1.15	−.15**	−.04*	.14**			
5. Interpersonal action	2.10	1.94	−.10*	.01	.14**	.15**		
6. Communal action	0.68	1.19	.06	.20***	.26***	.28***	.43***	
7. Political action	1.22	1.80	.07	.23***	.27***	.26***	.47***	.77***

*p < .05, **p < .01, ***p < .001.

**Table 2:** Standardized Model Effects for Critical Reflection

Variable	*B*	*SE*	*p*
Intercept	2.253	.560	<.001
Gender	0.064	.045	.16
Age	−0.029	.045	.52
Black	0.067	.045	.14
Equal status	−0.132	.053	.01
Cultural socialization	−0.261	.061	<.001
Critical consciousness socialization	0.508	.052	<.001

**Table 3:** Standardized Model Effects for Critical Action

Variable	Interpersonal Action			Communal Action			Political Action		
	*B*	*SE*	*p*	*B*	*SE*	*p*	*B*	*SE*	*p*
Intercept	−.090	.553	.87	−.384	.537	.48	−.403	.548	.46
Reflection	.157	.048	<.001	.284	.045	<.001	.273	.046	<.001
Gender	−.069	.049	.16	.041	.048	.39	−.003	.048	.95
Age	.081	.049	.10	.024	.048	.61	.013	.048	.78
Black	−.029	.049	.55	−.047	.048	.33	−.044	.048	.36
Cultural socialization							.105	.031	<.001

None of the covariates were significantly related to critical reflection or critical action. As expected, equal status was negatively correlated with critical reflection, such that adolescents who perceived more unfair treatment in their school were more likely to understand societal inequality. Furthermore, critical consciousness socialization (perceptions of opportunities to learn about racism) was associated with higher critical reflection. In contrast to our hypothesis, perceptions of opportunity to learn about one's own culture were negatively related to critical reflection. However, cultural socialization had an independent direct path to political action, such that higher perceptions were associated with more actions such as contacting an elected official.

In terms of mediation, critical reflection was positively related to each form of action. The bootstrapped indirect effects, reported in Table 4, show that critical reflection was a significant mediator for each path between racial climate and interpersonal, communal, and political action, which confirmed our hypothesis.

**Table 4:** Bootstrapped Indirect Effects of School Racial Climate on Critical Action through Critical Reflection

Indirect Effect	Confidence Interval Lower Level	Confidence Interval Higher Level
Equal status		
Interpersonal action	−.102	−.007
Communal action	−.088	−.008
Political action	−.129	−.013
Cultural socialization		
Interpersonal action	−.141	−.022
Communal action	−.137	−.044
Political action	−.204	−.057
Critical consciousness socialization		
Interpersonal action	.068	.310
Communal action	.115	.295
Political action	.160	.431

## DISCUSSION

The goal of the current study was to explore how school racial climate perceptions were associated with critical reflection and critical action. Specifically, our hypotheses about equal status and critical consciousness socialization were confirmed. Scholars have described how experiences with unfair treatment can alert youth to inequalities in society (Anyiwo et al., 2018; Hope et al., 2020; Mathews et al., 2019; Rubin, 2007; Tyler et al., 2020), and our study shows how seeing racial inequalities in one's immediate school context may lead youth to conclude that their school experiences represent systemic issues (Hope et al., 2015). In an analysis with this sample that also included Asian American and White students, Byrd and Ahn (2020) also found that a profile that experienced high discrimination from school, online, and neighborhoods also had the highest critical reflection and action. Reacting to inequality through civic engagement can be a healthy coping mechanism (Hope & Spencer, 2017; Hope, Gugwor, et al., 2019) that may offset the negative effects of racial discrimination.

We further saw that perceptions of teachers addressing racism and inequality in the curriculum (critical consciousness socialization) were also related to critical reflection (Anyiwo et al., 2018; Cammarota & Romero, 2011; Richards‐Schuster & Aldana, 2013; Seider et al., 2017). Our findings are in contrast to Bañales et al. (2021), who did not find a significant relation between critical consciousness socialization and critical reflection using the same measures. However, they did find that critical consciousness socialization was directly related to interpersonal and communal action, wherein in our study, we saw an indirect relation. The differences may be related to the fact that their sample was older (*M* = 17.00, *SD* = 1.29) and included White, Asian American, and Native American youth. Some studies suggest that Latinx youth pay more attention to racial dynamics and may be more strongly affected by them (Yip et al., 2019).

We also found a direct, positive relation between cultural socialization and political action above and beyond the effect of critical reflection. Curricula focused on historical events in a student's culture, like the Civil Rights Movement, may give youth models for effective political action and encourage their involvement. A surprising finding, however, was the negative relation between cultural socialization and critical reflection, which was seen in both the bivariate correlations and the path analysis. Learning about one's culture and being encouraged to show cultural pride have been related to positive academic outcomes when the messages come from parents or teachers (Byrd, 2017; Saleem et al., 2021; Wang et al., 2020). Furthermore, research supports greater critical consciousness when schools have a diverse and open curriculum (Bañales et al., 2019; Godfrey & Grayman, 2014; Seider et al., 2020). However, little research has investigated how a focus on one culture (i.e., the students' background) prompts or inhibits critical reflection. Since the analysis controls for critical consciousness socialization, it may be that cultural pride messages in isolation from messages about inequality may prompt youth to believe that society is fair and equal. In a qualitative study of African American adolescents, the youth discussed exposure to contemporary Black figures such as Barack Obama and Ben Carson but had little understanding of structural racism because of a lack of critical consciousness socialization (Byrd & Hope, 2020).

Importantly, in our study critical reflection was a significant mediator of the relation between school racial socialization messages and critical action, which suggests that our Black and Latinx youths' actions were explained in part by their increasing awareness of inequality in society (Diemer et al., 2016; Singh et al., 2021). Previous research has supported the idea of the school context as an important space for the development of critical consciousness (Heberle et al., 2020; Seider et al., 2017, 2020) and has found that critical reflection functions as a mediator between adolescents' experiences and critical action (Hope et al., 2020; Tyler et al., 2020). Our study has shown two areas of the school context that are important: discriminatory interactions and messages in the curriculum discussing racial inequality. Youths' thinking about their experiences with racial discrimination can prompt them to consider that their interactions are not simply random occurrences but are based on historical patterns (Mathews et al., 2019. Furthermore, by including content about politics, historical events, and controversial topics, teachers can help youth understand the structural nature of disparities between ethnic–racial groups (Godfrey & Grayman, 2014).

The implications of this finding are that educators and parents should be vigilant against calls to reduce or eliminate the teaching of history and current events involving race and racism. For example, in North Carolina, a task force led by the Lieutenant Governor encouraged parents and teachers to “hold the system accountable” by submitting examples of “indoctrination” and “inappropriate content” in K‐12 schools in conjunction with statewide and national efforts to restrict anti‐racist teaching (“F.A.C.T.S. | Lieutenant Governor”, n.d.; Stout & Wilburn, 2022). If such efforts are successful, either by legislating the curriculum or by creating a climate in which teachers are reluctant to discuss such issues, a vital pathway for youths' critical contributions to society is foreclosed. Research on critical consciousness development is necessary for educators to understand just how essential their work in classrooms is for dismantling racism and oppression. For researchers, further work is necessary to explore the precise mechanisms through which youths' perceptions translate into beliefs about inequality and the desire for critical action.

## LIMITATIONS AND FUTURE DIRECTIONS

A limitation of this study was that it was cross‐sectional; therefore, we were not able to determine causality. Critical reflection or involvement in critical action influences school racial socialization perceptions. For example, the youth who are most aware of inequality in society may be primed to look for inequality within their schools, such that they are more likely to interpret negative cross‐race interactions as unfair discrimination. These students may also be more likely to seek out lessons about their own cultures at school and judge the school negatively when they do not see them. Some scholars feel that cross‐sectional data are not appropriate for tests of mediation; therefore, longitudinal studies are essential for exploring the dynamics of critical consciousness development. Furthermore, a person‐centered approach (e.g., Byrd & Ahn, 2020) may have better represented the multidimensional nature of school racial climate perceptions. Some students perceive positive interracial interactions and positive school racial socialization, some students perceive negative interracial interactions and a lack of school racial socialization, whereas other students experience positive interracial interactions with low socialization, and these differing profiles may have unique implications for critical reflection and critical action.

School contexts are complex, and research should do more to consider how youth negotiate messages from multiple sources. Work on familial socialization may serve as a guide. For instance, A study of African American and Latinx adolescents found that congruent cultural socialization messages from family and peers resulted in better socioemotional and academic outcomes (Wang et al., 2020). However, unlike familial socialization, school socialization comes with many more sources: teachers and staff, peers, and institutional practices and policies (Saleem & Byrd, 2021). Future research should consider the process of integration in racial socialization research, for example, by considering how the frequency or timing of messages is associated with its relation to adolescents' developing beliefs about inequality.

In conclusion, this study has indicated that negative interracial interactions and opportunities to learn about structural racism can help youth to understand structural racism and promote critical action. Researchers and educators should work to understand the ways in which all dimensions of critical consciousness can be fostered in adolescents. Dismantling oppression can begin with resistance in the classroom: through teachers raising students' awareness of histories of racism and the ways in which racism occurs in their own school setting. For many youth, this increased awareness will prompt them to take on oppression as it manifests in their individual interactions, their communities, and society at large.

## References

[jora12778-bib-0001] Aldana, A. , Bañales, J. , & Richards‐Schuster, K. (2019). Youth anti‐racist engagement: Conceptualization, development, and validation of an anti‐racism action scale. Adolescent Research Review, 4(4), 369–381.

[jora12778-bib-0002] Anyiwo, N. , Bañales, J. , Rowley, S. J. , Watkins, D. C. , & Richards‐Schuster, K. (2018). Sociocultural influences on the sociopolitical development of African American youth. Child Development Perspectives, 12(3), 165–170.

[jora12778-bib-0003] Bañales, J. , Aldana, A. , Richards‐Schuster, K. , Flanagan, C. A. , Diemer, M. A. , & Rowley, S. J. (2019). Youth anti‐racism action: Contributions of youth perceptions of school racial messages and critical consciousness. Journal of Community Psychology, 49, 3079–3100. 31691984 10.1002/jcop.22266

[jora12778-bib-0004] Bañales, J. , Hope, E. C. , Rowley, S. J. , & Cryer‐Coupet, Q. R. (2021). Raising justice‐minded youth: Parental ethnic‐racial and political socialization and Black youth's critical consciousness. Journal of Social Issues, 77(4), 964–986. 10.1111/josi.12486

[jora12778-bib-0005] Bañales, J. , Marchand, A. D. , Skinner, O. D. , Anyiwo, N. , Rowley, S. J. , & Kurtz‐Costes, B. (2020). Black adolescents' critical reflection development: Parents' racial socialization and attributions about race achievement gaps. Journal of Research on Adolescence, 30, 403–417. 30758108 10.1111/jora.12485

[jora12778-bib-0006] Byrd, C. M. (2015). The associations of intergroup interactions and school racial socialization with academic motivation. The Journal of Educational Research, 108(1), 10–21.

[jora12778-bib-0007] Byrd, C. M. (2017). The complexity of school racial climate: Reliability and validity of a new measure for secondary students. British Journal of Educational Psychology, 87(4), 700–721. 10.1111/bjep.12179 28850714

[jora12778-bib-0008] Byrd, C. M. , & Ahn, L. H. (2020). Profiles of ethnic‐racial socialization from family, school, neighborhood, and the Internet: Relations to adolescent outcomes. Journal of Community Psychology, 48(6), 1942–1963. 10.1002/jcop.22393 32526066

[jora12778-bib-0009] Byrd, C. M. , & Hope, E. C. (2020). Black students' perceptions of school ethnic‐racial socialization practices in a predominantly Black school. Journal of Adolescent Research, 35(6), 728–753. 10.1177/0743558419897386

[jora12778-bib-0010] Cammarota, J. , & Romero, A. (2011). Participatory action research for high school students: Transforming policy, practice, and the personal with social justice education. Educational Policy, 25(3), 488–506. 10.1177/0895904810361722

[jora12778-bib-0011] Diemer, M. A. , Rapa, L. J. , Park, C. J. , & Perry, J. C. (2017). Development and validation of the critical consciousness scale. Youth & Society, 49(4), 461–483.

[jora12778-bib-0012] Diemer, M. A. , Rapa, L. J. , Voight, A. M. , & McWhirter, E. H. (2016). Critical consciousness: A developmental approach to addressing marginalization and oppression. Child Development Perspectives, 10(4), 216–221. 10.1111/cdep.12193

[jora12778-bib-0013] F.A.C.T.S. | Lieutenant Governor . (n.d.). https://ltgov.nc.gov/facts

[jora12778-bib-0014] Flanagan, C. , & Levine, P. (2010). Civic engagement and the transition to adulthood. The Future of Children, 20, 159–179. 20364626 10.1353/foc.0.0043

[jora12778-bib-0015] Freire, P. (1970). Pedagogy of the oppressed. Continuum.

[jora12778-bib-0016] Godfrey, E. B. , & Grayman, J. K. (2014). Teaching citizens: The role of open classroom climate in fostering critical consciousness among youth. Journal of Youth and Adolescence, 43(11), 1801–1817. 24395151 10.1007/s10964-013-0084-5

[jora12778-bib-0017] Golden, A. R. , Anderson, R. E. , Cooper, S. M. , Hope, E. C. , & Kloos, B. (2021). “With politics, it's easier to talk to them about it”: Peer racial socialization and sociopolitical development among black college students. Emerging Adulthood. 10.1177/21676968211040321

[jora12778-bib-0018] Golden, A. R. , Griffin, C. B. , Metzger, I. W. , & Cooper, S. M. (2018). School racial climate and academic outcomes in African American adolescents: The protective role of peers. Journal of Black Psychology, 44(1), 47–73. 10.1177/0095798417736685

[jora12778-bib-0019] Gopalan, M. , & Nelson, A. A. (2019). Understanding the racial discipline gap in schools. AERA Open, 5(2), 2332858419844613.

[jora12778-bib-0020] Hayes, A. F. (2018). Introduction to mediation, moderation, and conditional process analysis: A regression‐based approach. Guilford Publications.

[jora12778-bib-0021] Heberle, A. E. , Rapa, L. J. , & Farago, F. (2020). Critical consciousness in children and adolescents: A systematic review, critical assessment, and recommendations for future research. Psychological Bulletin, 146(6), 525–551. 10.1037/bul0000230 32271028

[jora12778-bib-0022] Hope, E. C. , & Bañales, J. (2019). Black early adolescent critical reflection of inequitable sociopolitical conditions: A qualitative investigation. Journal of Adolescent Research, 34(2), 167–200.

[jora12778-bib-0023] Hope, E. C. , Gugwor, R. , Riddick, K. N. , & Pender, K. N. (2019). Engaged against the machine: Institutional and cultural racial discrimination and racial identity as predictors of activism orientation among Black youth. American Journal of Community Psychology, 63(1–2), 61–72. 30659621 10.1002/ajcp.12303

[jora12778-bib-0024] Hope, E. C. , Hoggard, L. S. , & Thomas, A. (2015). Emerging into adulthood in the face of racial discrimination: Physiological, psychological, and sociopolitical consequences for African American youth. Translational Issues in Psychological Science, 1(4), 342–351.

[jora12778-bib-0025] Hope, E. C. , Pender, K. N. , & Riddick, K. N. (2019). Development and validation of the Black community activism orientation scale. Journal of Black Psychology, 45(3), 185–214.

[jora12778-bib-0026] Hope, E. C. , Smith, C. D. , Cryer‐Coupet, Q. R. , & Briggs, A. S. (2020). Relations between racial stress and critical consciousness for black adolescents. Journal of Applied Developmental Psychology, 70, 101184.

[jora12778-bib-0027] Hope, E. C. , & Spencer, M. B. (2017). Civic engagement as an adaptive coping response to conditions of inequality: An application of phenomenological variant of ecological systems theory (PVEST). In Handbook on positive development of minority children and youth (pp. 421–435). Springer.

[jora12778-bib-0028] Jemal, A. (2017). Critical consciousness: A critique and critical analysis of the literature. The Urban Review, 49(4), 602–626. 29657340 10.1007/s11256-017-0411-3 PMC5892452

[jora12778-bib-0029] Johnston‐Goodstar, K. , & VeLure Roholt, R. (2017). “Our kids aren't dropping out; they're being pushed out”: Native American students and racial microaggressions in schools. Journal of Ethnic & Cultural Diversity in Social Work, 26(1–2), 30–47. 10.1080/15313204.2016.1263818

[jora12778-bib-0030] Kaufman, J. S. , Jaser, S. S. , Vaughan, E. L. , Reynolds, J. S. , Di Donato, J. , Bernard, S. N. , & Hernandez‐Brereton, M. (2010). Patterns in office referral data by grade, race/ethnicity, and gender. Journal of Positive Behavior Interventions, 12(1), 44–54. 10.1177/1098300708329710 25580076 PMC4286304

[jora12778-bib-0031] Kelly, L. L. (2018). A snapchat story: How black girls develop strategies for critical resistance in school. Learning, Media and Technology, 43(4), 374–389. 10.1080/17439884.2018.1498352

[jora12778-bib-0032] Leath, S. , & Chavous, T. (2017). “We really protested”: The influence of sociopolitical beliefs, political self‐efficacy, and campus racial climate on civic engagement among Black college students attending predominantly White institutions. The Journal of Negro Education, 86(3), 220–237.

[jora12778-bib-0033] Leath, S. , Mathews, C. , Harrison, A. , & Chavous, T. (2019). Racial identity, racial discrimination, and classroom engagement outcomes among Black girls and boys in predominantly Black and predominantly White school districts. American Educational Research Journal, 56(4), 1318–1352.

[jora12778-bib-0034] Lozada, F. T. , Jagers, R. J. , Smith, C. D. , Bañales, J. , & Hope, E. C. (2017). Prosocial behaviors of Black adolescent boys: An application of a sociopolitical development theory. Journal of Black Psychology, 43(5), 493–516. 10.1177/0095798416652021

[jora12778-bib-0035] Mathews, C. J. , Medina, M. A. , Bañales, J. , Pinetta, B. J. , Marchand, A. D. , Agi, A. C. , Miller, S. M. , Hoffman, A. J. , Diemer, M. A. , & Rivas‐Drake, D. (2019). Mapping the intersections of adolescents' ethnic‐racial identity and critical consciousness. Adolescent Research Review, 5, 1–17.

[jora12778-bib-0036] Matthews, J. S. , & López, F. (2019). Speaking their language: The role of cultural content integration and heritage language for academic achievement among Latino children. Contemporary Educational Psychology, 57, 72–86. 10.1016/j.cedpsych.2018.01.005

[jora12778-bib-0037] Maxwell, S. E. , Cole, D. A. , & Mitchell, M. A. (2011). Bias in cross‐sectional analyses of longitudinal mediation: Partial and complete mediation under an autoregressive model. Multivariate Behavioral Research, 46(5), 816–841. 10.1080/00273171.2011.606716 26736047

[jora12778-bib-0038] Pinedo, A. , Durkee, M. I. , Diemer, M. A. , & Hope, E. C. (2021). Disentangling longitudinal trajectories of racial discrimination and critical action among Black and Latinx college students: What role do peers play? Cultural Diversity and Ethnic Minority Psychology, 27, 546–557. 33914581 10.1037/cdp0000434

[jora12778-bib-0039] Richards‐Schuster, K. , & Aldana, A. (2013). Learning to speak out about racism: Youths' insights on participation in an intergroup dialogues program. Social Work with Groups, 36(4), 332–348. 10.1080/01609513.2013.763327

[jora12778-bib-0040] Rubin, B. C. (2007). There's still not justice: Youth civic identity development amid distinct school and community contexts. Teachers College Record, 109(2), 449–481.

[jora12778-bib-0041] Ruck, M. D. , & Wortley, S. (2002). Racial and ethnic minority high school students' perceptions of school disciplinary practices: A look at some Canadian findings. Journal of Youth and Adolescence, 31(3), 185–195.

[jora12778-bib-0042] Saleem, F. T. , & Byrd, C. M. (2021). Unpacking school ethnic‐racial socialization: A new conceptual model. Journal of Social Issues, 77(4), 1106–1125.

[jora12778-bib-0043] Seider, S. , Kelly, L. , Clark, S. , Jennett, P. , El‐Amin, A. , Graves, D. , Soutter, M. , Malhotra, S. , & Cabral, M. (2020). Fostering the sociopolitical development of African American and Latinx adolescents to analyze and challenge racial and economic inequality. Youth & Society, 52(5), 756–794. 10.1177/0044118X18767783

[jora12778-bib-0044] Seider, S. , Tamerat, J. , Clark, S. , & Soutter, M. (2017). Investigating adolescents' critical consciousness development through a character framework. Journal of Youth and Adolescence, 46(6), 1162–1178. 10.1007/s10964-017-0641-4 28210923

[jora12778-bib-0045] Singh, S. , Berezin, M. N. , Wallach, L. N. , Godfrey, E. B. , & Javdani, S. (2021). Traumatic incidents and experiences of racism and sexism: Examining associations with components of critical consciousness for system‐involved girls of color. American Journal of Community Psychology, 67(1–2), 64–75. 10.1002/ajcp.12479 33249601

[jora12778-bib-0046] Stout, C. , & Wilburn, T. (2022). CRT MAP: Critical race theory legislation and schools. https://www.chalkbeat.org/22525983/map‐critical‐race‐theory‐legislation‐teaching‐racism

[jora12778-bib-0047] Stowe, R. (2017). Culturally responsive teaching in an Oglala Lakota classroom. The Social Studies, 108(6), 242–248.

[jora12778-bib-0048] Tyler, C. P. , Olsen, S. G. , Geldhof, G. J. , & Bowers, E. P. (2020). Critical consciousness in late adolescence: Understanding if, how, and why youth act. Journal of Applied Developmental Psychology, 70, 101165. 10.1016/j.appdev.2020.101165 PMC7443156 32863511

[jora12778-bib-0049] Wang, Y. , Benner, A. D. , & Boyle, A. E. (2020). Family cultural socialization in childhood: Navigating ethnic/racial diversity and numeric marginalization in school and neighborhood settings. Cultural Diversity and Ethnic Minority Psychology. Advance online publication. 10.1037/cdp0000435 PMC8441026 33370137

[jora12778-bib-0050] Watts, R. J. , Diemer, M. A. , & Voight, A. M. (2011). Critical consciousness: Current status and future directions. New Directions for Child and Adolescent Development, 2011(134), 43–57. 10.1002/cd.310 22147600

[jora12778-bib-0051] Watts, R. J. , & Flanagan, C. (2007). Pushing the envelope on youth civic engagement: A developmental and liberation psychology perspective. Journal of Community Psychology, 35(6), 779–792.

[jora12778-bib-0052] Watts, R. J. , Griffith, D. M. , & Abdul‐Adil, J. (1999). Sociopolitical development as an antidote for oppression—Theory and action. American Journal of Community Psychology, 27(2), 255–271.

[jora12778-bib-0053] Wegmann, K. M. , & Smith, B. (2019). Examining racial/ethnic disparities in school discipline in the context of student‐reported behavior infractions. Children and Youth Services Review, 103, 18–27.

[jora12778-bib-0054] White‐Johnson, R. L. (2012). Prosocial involvement among African American young adults: Considering racial discrimination and racial identity. Journal of Black Psychology, 38(3), 313–341.

[jora12778-bib-0056] Woodson, A. N. (2015). “What you supposed to know”: Urban black students’ perspectives on history textbooks. Journal of Black Psychology, 11, 57–55.

[jora12778-bib-0055] Yip, T. , Wang, Y. , Mootoo, C. , & Mirpuri, S. (2019). Moderating the association between discrimination and adjustment: A meta‐analysis of ethnic/racial identity. Developmental Psychology, 55(6), 1274–1298. 30907605 10.1037/dev0000708 PMC6557142

